# Supplementary material for: Heart Failure Therapies for the Prevention of HER2-Monoclonal Antibody-Mediated Cardiotoxicity: A Systematic Review and Meta-Analysis of Randomized Trials
Source: Cancers (Basel). 2021 Nov 3;13(21):5527. doi: 10.3390/cancers13215527 (PMC8583665; doi:10.3390/cancers13215527)
Supplement: Supplementary file 1 [file cancers-13-05527-s001.zip › cancers-1419681-supplementary.pdf]

Supplementary Material

Supplementary Table S1: Search Strategy and Results

Data Source				Limits				Duplicates Results			
Database	Vendor	Date searched	Database update	English only?	Time Period Searched	Publication types	Other Limits	Items found	Inner dups	Ext dups	New
Medline/PubMed	Ovid	12/8/21	-	No	No limit	No limit	-	175	0	0	175
Query: ((breast cancer) AND ((trastuzumab*) OR (pertuzumab))) AND ((prevention) AND (cardiotoxicity))											
EMBASE	Elsevier	12/8/21	-	No	No limit	No limit	-	703	6	25	672
Query: (breast cancer.mp. or exp breast cancer/ AND (exp trastuzumab/ or trastuzumab.mp. OR exp pertuzumab/ or pertuzumab.mp.) AND (prevention.mp. or exp prevention/OR cardiotoxicity.mp. or exp cardiotoxicity/)											
Scopus	Elsevier	12/8/21	-	No	No limit	Article	-	1653	2	105	1546
Query: (((ALL ("breast cancer")) AND (ALL ("trastuzumab*") OR ALL ("pertuzumab"))) AND (ALL ("prevention") AND ALL ("cardiotoxicity"))) AND (LIMIT-TO (DOCTYPE , "ar"))											
Cochrane Library	Wiley	12/8/21	-	No	No limit	No limit	-	54	2	20	32
Query: "cardiotoxicity" AND "prevention" in All Text AND "breast cancer" AND ("trastuzumab" OR "pertuzumab") in All Text - (Word variations have been searched)											

	Items found	Inner dups	Ext dups	New
Totals	2585	10	150	2425

Supplementary Table S2: Summary of Findings Table

Certainty assessment							Summary of findings				
							Study event rates (%)		Relative effect (95% CI)	Anticipated absolute effects	
Participants (studies) Follow-up	Risk of bias	Inconsistency	Indirectness	Imprecision	Publication bias	Overall certainty of evidence	With placebo	With heart failure therapies		Risk with placebo	Risk difference with heart failure therapies
**Cardiotoxicity**											
952 (3 RCTs)	not serious	serious[a]	not serious	serious[b]	none	⊕⊕◯◯ Low	120/471 (25.5%)	110/481 (22.9%)	**RR 0.90** (0.63 to 1.29)	255 per 1,000	**25 fewer per 1,000** (from 94 fewer to 74 more)
**Follow-up LVEF**											
466 (4 RCTs)	serious[c]	serious[a]	not serious	not serious	none	⊕⊕◯◯ Low	236	230	-	The mean follow-up LVEF ranged from **54.5-59.3** %	MD **2.24 % higher** (0.53 higher to 3.94 higher)
**Trastuzumab interruption**											
740 (2 RCTs)	not serious	not serious	not serious	not serious	none	⊕⊕⊕⊕ High	98/364 (26.9%)	57/376 (15.2%)	**RR 0.57** (0.43 to 0.77)	269 per 1,000	**116 fewer per 1,000** (from 153 fewer to 62 fewer)

CI: confidence interval; **MD:** mean difference; **RR:** risk ratio

Explanations

a. Moderate unexplained heterogeneity
b. Confidence interval fails to exclude significant benefit or harm
c. Two included studies (Farahani et al. and Sherafati et al.) had 'some concerns' on RoB2 assessment, in one domain each

Supplementary Table S3: Risk of Bias Table

Study ID	Experimental	Comparator	Outcome	Weight	D1	D2	D3	D4	D5	Overall
Boekhoet 2016	Candesartan	Placebo	Primary	1	+	+	+	+	+	+
Farahani 2019	Carvedilol	No Therapy	Primary	1	+	!	+	+	+	!
Guglin 2019	Carvidelol/Lisopril	Placebo	Primary	1	+	+	+	+	+	+
Pitushkin 2017	Bisoprolol/Perindopril	Placebo	Primary	1	+	+	+	+	+	+
Sherafati 2018	Carvedilol	No Therapy	Primary	1	+	+	+	+	!	!

+	Low risk
!	Some concerns
-	High risk

D1	Randomisation process
D2	Deviations from the intended interventions
D3	Missing outcome data
D4	Measurement of the outcome
D5	Selection of the reported result
